# The Response of Critical Microbial Taxa to Litter Micro-Nutrients and Macro-Chemistry Determined the Agricultural Soil Priming Intensity After Afforestation

**DOI:** 10.3389/fmicb.2021.730117

**Published:** 2021-09-16

**Authors:** Hongling Yang, Yulin Li, Shaokun Wang, Jin Zhan, Zhiying Ning, Dan Han

**Affiliations:** ^1^Naiman Desertification Research Station, Northwest Institute of Eco-Environment and Resources, Chinese Academy of Sciences (CAS), Lanzhou, China; ^2^College of Resources and Environment, University of Chinese Academy of Sciences, Beijing, China; ^3^Urat Desert-Grassland Research Station, Northwest Institute of Eco-Environment and Resources, Chinese Academy of Sciences (CAS), Lanzhou, China

**Keywords:** afforestation, agro-forest-grass composite ecosystem, soil priming effect, litter chemical traits, critical microbial taxa, macro-chemistry

## Abstract

Afforestation with trees and shrubs around cropland can effectively decrease soil degradation and avoid sand storms, but subsequent modification of litter quality accelerates the degradation of native organic matter *via* the soil priming effect (PE). Although carbon accumulation in agricultural soils after afforestation was widely studied, little is known about the extent to which soil organic carbon (SOC) mineralization is induced by complex residue input in agro-forest-grass composite ecosystems. Here, we mixed corn field soil and litter of afforestation tree and shrub species together in a micro-environment to quantify the effects of litter-mixture input on farmland soil priming associated with afforestation. Additionally, we studied the responses of bacterial and fungal species to litter chemistry, with the aim to identify the litter and microbial driver of soil priming. The results showed that soil priming was accelerated by different litter addition which varied from 24 to 74% of SOC mineralization, suggesting that priming intensity was relatively flexible and highly affected by litter quality. We also find that the macro-chemistry (including litter carbon, nitrogen, lignin, and cellulose) directly affects priming intensity, while micro-chemistry (including litter soluble sugar, water-soluble phenol, methanol-soluble phenol, and condensed tannin) indirectly influences priming *via* alteration to dominant bacterial taxa. The stepwise regression analysis suggested that litter nitrogen and cellulose were the critical litter drivers to soil priming (*r*^2^ = 0.279), and the combination of bacterial phylum *Proteobacteria*, *Firmicutes*, *Bacteroidetes*, *Acidobacteria*, and fungal taxa *Eurotiomycetes* was a great model to explain the priming intensity (*r*^2^ = 0.407).

## Introduction

Afforestation (artificial planting of trees or shrubs) is believed to be one of the most effective ways to restore degraded soils and to sequester carbon in northern farming-pastoral ecotones (Russell et al., 2005; Hernandez-Ramirez et al., 2011; Li et al., 2017). It has been estimated that 6.42 × 10^3^ km^2^ of farmland was planted with trees and shrubs and 26% of soil organic carbon (SOC) stocks was increased in such a fragile ecological environment (Laganière et al., 2010; Mengzhu et al., 2021). However, modification of litter quality accompanied by afforestation also stimulates mineralization of native SOC called priming effect (PE) (Kuzyakov et al., 2000; Chenu et al., 2018; Guenet et al., 2018). A meta-analysis at global scale has demonstrated that priming accounts for 47.5% of native SOC mineralization which means SOC degradation across different ecosystems (Sun et al., 2019). To get a knowledge of soil carbon status of tree and shrub plantations, it is therefore imperative to quantify to what extent SOC mineralization is accelerated by inputs of litter mixtures in converted farmland.

Priming is ubiquitous in different ecosystems, but exhibits high variability from 14 to 380% of native SOC loss (Luo, 2015; Huo et al., 2017). Priming is driven by multiple mechanisms involving abiotic and biotic factors such as soil properties, substrate quality, and microbial activity (Hicks et al., 2019; Liu X.J.A. et al., 2020). Recent evidence suggests that residue quality may be an important determinant of priming (Bastida, 2019; Chen et al., 2019b). Most studies of potential priming mechanisms used labeled simple C-compounds or biochar as surrogates of plant inputs (Conde et al., 2005; Hamer and Marschner, 2005; Chen et al., 2019a), however, glucose-like C or biochar are unable to represent the complex C structures of litter residues. In fact, residue quality in natural ecosystems is a broad term that includes macro-chemistry variables such as C and N contents and micro-chemistry like phenols and condensed tannin (Ct) concentration, which are mostly represented by the ratio of N to lignin in current studies. For example, the Microbial Efficiency-Matrix Stabilization (MEMS) hypothesis states that litters with high N and low lignin content contribute to significant soil organic matter stabilization in the mineral soil matrix (Cotrufo et al., 2013). This has recently been challenged by studies demonstrating that high-quality substrate (high N/lignin) is expected to prime the loss of the original carbon source more efficiently than of the added one (Blagodatskaya et al., 2014; Lyu et al., 2019). To some extent, this inconsistency can be attributed to the fact that most of the research on litter quality did not include micro chemical compounds, such as tannins (Shahbaz et al., 2018; Sun et al., 2019). It has been shown that the magnitude of priming was much higher in residues containing large amounts of tannins than in those with low tannin contents (Shahbaz et al., 2018). Consequently, the role of micronutrients in accelerated mineralization of soil organic matter should be considered.

Additionally, intensity of the PE may be due to, at least in part, the direct response of soil microbial community to added fresh organic matter, since most soil microbes are considered energy- or easily-available substrates-limited (Derrien et al., 2014). It has been reported that a supply of high-quality litter contributed to a large amount of labile carbon and nitrogen, and that microbes preferentially utilized these labile organic carbon sources (Kuzyakov et al., 2000). In contrast, when litter with low nitrogen was added, microbes switched to soil organic matter to obtain energy and nutrients (Murphy et al., 2015). Traditional explanations focused on microbial biomass and community composition underlying the PE, it is unclear whether specific microbes are responsible for soil organic matter mineralization (Kim et al., 2015). However, different microbial taxa differ in their roles in the mineralization and assimilation of litter and soil-derived C (Chen et al., 2019a). It has been shown that bacteria are the first to mineralize labile C entering the soil, while fungi usually feed on complex organic compounds (Razanamalala et al., 2018). Beyond this, the role of specific microbial groups in accelerated mineralization of soil organic matter is poorly elucidated.

Over the last 40 years, large-scale *Populus simonii* afforestation and *Artemisia halondendron* and *Caragana microphylla* shelterbelt establishment projects have been conducted in China to avoid soil desertification in China’s northern farming-pastoral ecotones (Li et al., 2017; Hong et al., 2018). Afforestation and shelterbelt establishment effectively promoted soil and water protection and soil new C storage (Zeng et al., 2009), but introduced a new problem: tree and shrub litter mixture was blown by strong winds to lowland cornfields and then soil microbes were activated to utilize litter and soil organic matter causing farmland soil PE.

The primary objective of this study was therefore to investigate the effect of inputs of diverse tree and shrub leaf mixtures on SOC mineralization in farmland. To achieve this goal, we incubated farmland soil, litter from *P. simonii*, *A. halondendron*, and *C. microphylla* in a laboratory experiment to simulate a natural farmland ecosystem. We hypothesized that (a) complex litter mixture addition causes the loss of SOC through priming in an agro-forest-grass composite ecosystem, (b) litter macro- and micro-nutrients directly influence the magnitude of priming, and (c) some specific bacterial and fungal taxa are responsible for the soil PE. We measured bacterial community composition, SOC mineralization, released C from litter, and we calculated PE and priming efficiency based on the natural abundance difference in δ^13^C values between farmland soil and litter. We further analyzed the effect of litter chemistry and dominant microbial taxa on soil processes with the purpose of identifying the key factor that influences soil PE.

## Materials and Methods

### Soil and Litter Sampling, and Incubation

The soil used in the incubation experiment was sampled from the plow layer of dryland agricultural areas at the Naiman Desertification Research Station, Chinese Academy of Sciences in eastern part of the agro-grazing ecotone in China (N42°55′ and E120°41′, 350 m above sea level). This site was widely cultivated with C_4_ maize crop, but over the last 40 years, this region has undergone severe desertification (Ning et al., 2021). Many agroforestry practices have been conducted to counteract soil erosion and reduce land degradation in this area. To be specifically, *C. microphylla* and *A. halondendron* are deciduous shrub that was widely planted because of its high tolerance against drought, wind erosion, and sand burial (Luo et al., 2019; Wang et al., 2020).

The long-term mean annual precipitation is 343 mm and annual temperature at the site is 6.4°C, with nearly 80% falling between May and September (Luo et al., 2019). The mean annual wind speed ranges from 3.4 to 4.1 m s^–1^ (Wang et al., 2020). The δ^13^C signature of maize soil was −16.5‰. Soil pH was 7.8, and the maximum water holding capacity was 14.9%.

Land use in the northeastern agro-grazing ecotone is characterized by farmland wind break tree belts of *A. halondendron* and *C. microphylla* with scattered *P. simonii* trees (Li et al., 2016). In September 2017, litters of *Artemisia halodendron* (Ah), *C. microphylla* (Cm), and *P. simonii* (Ps) were collected and stored in laboratory. We only sampled leaf material that was freshly senesced, still attached to plant and was not attacked by microbes. On April 28, 2018, the stored litter sample was oven-dried at 65°C for 24 h for further incubation experiment, that was mainly because plant litters generally varied in their water content due to difference in litter structure and chemical composition, which may lead to great difference in the dry weight when we added same amount litter to soil. To simulate a natural composite ecosystem in agro-grazing ecotone, different litter species were mixed in equal proportions and therefore there were seven litter addition treatments (Ah, Cm, Ps, AhCm, AhPs, CmPs, and AhCmPs). Two grams of litter mixture, corresponding to a natural yearly quantity of litter in this region (Xuyang et al., 2018) were then ball-milled into powder (GT300 Ball Mill, POWTEQ, Beijing, China) to minimize the influence of size and structure of litter material since we were more interested in the role of litter chemistry. Then the litter powder was homogenized by passing through 0.25-mm-mesh sieve to avoid aeration or channeling of the soil induced by litter addition. Because 88.2% of soil particle was greater than 0.25 mm, the soil porosity induced by litter addition was hence cab be negligible. There were five replicates in each leaf litter treatment.

Litter powder was added to 200-g dry-weight soil and thoroughly mixed in 500-mL canning jar, and five replicates were control samples without litter input. Litter-soil mixture were maintained at 60% water-holding capacity through gravimetric method and were kept at 21–25°C in incubator (SPX-500; Jiangnan, Ningbo, China) for 111 days.

### Method Theory and Priming Calculation

We used the natural carbon abundance difference in δ^13^C values between litter of C_3_ plant and soil cultivated C_4_ maize for more than 30 years to separate litter-released carbon from SOC mineralization; this method is an effective natural tracer of C dynamics (Balesdent et al., 1987). We followed the variability in δ^13^C natural isotope abundance in soil-litter mixtures before and after incubation to allocate the litter-released C and the native SOC mineralization. Native C-SOM decomposition was calculated using Eqs 1–4:


(1) $$T\,O\,C_{F\,\text{inal}}=S\,O\,C_{F\,\text{inal}}+C_{L\,i\,t\,t\,e\,r}$$

where TOC_*Final*_ is the total carbon content at the end of the experiment, C_*Litter*_ is the C remaining in soil from the added litter, SOC_*Final*_ is the remaining native SOC. From Eq. 1 and the isotopic composition of litters and SOM, we obtained Eq. 2:


(2) $$\delta^{13}\,C_{F\,\text{inal}}\,{\mathrm{TOC}}_{F\,\text{inal}}=\delta^{13}\,C_{N\,\text{ative}-\mathrm{SOC}}\,{\mathrm{SOC}}_{F\,\text{inal}}+\delta^{13}\,C_{\mathrm{Litter}}\,C_{\mathrm{Litter}}$$

where δ^13^C_*Final*_ is the δ^13^C of the total organic carbon (soil-litter mixture) at the end of the experiment, δ^13^C_*Native*–*SOC*_ is the δ^13^C of the whole soil before the experiment, δ^13^C_*Litter*_ is the δ^13^C of litter without any change during incubation. Based on Eqs 1, 2, we calculated the portion of C resulting from plant litter (Eq. 3):


(3) $$C_{L\,\text{itter}}=\frac{T\,O\,C_{F\,\text{inal}}*\left(\delta^{13}\,C_{N\,\text{ative}-S\,O\,C}-\delta^{13}\,C_{F\,\text{inal}}\right)}{\delta^{13}\,C_{N\,\text{ative}-S\,O\,C}-\delta^{13}\,C_{L\,\text{itter}}}$$

Based on Eq. 1, we determined the remaining native SOC (SOC_*Final*_) and SOC loss resulting from SOC_*Initial*_ minus SOC_*Final*_. Therefore, the PE was the difference between native SOC loss in amended versus the non-amended treatment. The PE was calculated as follows:


(4) $$\mathrm{Priming}\,\mathrm{effect}={\left(S\,O\,C_{I\,\text{nitial}}-S\,O\,C_{F\,\text{ina}}\right)}_{T\,r\,e\,a\,t\,m\,e\,n\,t}-{\left(S\,O\,C_{I\,\text{nitial}}-S\,O\,C_{F\,\text{ina}}\right)}_{C\,o\,n\,t\,r\,o\,l}$$

Priming efficiency (%) was the increase in decomposition of native soil organic matter per unit plant litter added.

As mentioned above, SOC mineralization resulting from SOC_*Initial*_ minus SOC_*Final*_, thus we get Eq. 5


(5) $$S\,O\,C\,m\,i\,n\,e\,r\,a\,l\,i\,z\,a\,t\,i\,o\,n=S\,O\,C_{I\,n\,i\,i\,t\,i\,a\,l}-S\,O\,C_{F\,i\,n\,a\,l}$$

### Soil Carbon and Litter Chemistry Measurements

Soil was analyzed for SOC and δ^13^C. As we mentioned above, the soils in the study area are sandy with a coarse texture and loose structure, soil eluviation is serious and soil inorganic carbon in topsoil is only 0.018–0.15 g⋅kg^–1^ (Ning et al., 2021). Thus, soil total carbon content determined by the elemental analyzer represents the SOC content here. Organic C and δ^13^C of soil before and after incubation, and Organic C and δ^13^C of litters were analyzed with the element analyzer (Costech ECS 4010) coupled with an isotope analyzer (Picarro CM-CRDS). Litter nitrogen concentration was analyzed with an element analyzer (Costech ECS 4010). Lignin and cellulose concentrations were analyzed using a modified acetyl bromide method (Iiyama and Wallis, 1990) and an acid-hydrolysis method (Updegraff, 1969), respectively. Concentrations of water-soluble phenol (phW) and methanol-soluble phenol (phM) were determined with the Folin–Ciocalteu method (Waterman and Mole, 1998). Ct were measured according to the acid butanol method as described by Hagerman (Porter et al., 1986). Soluble sugar (Ss) concentration was determined using the anthrone method (Helbert and Brown, 2002). These chemical traits were chosen because of their demonstrated effects on litter decomposition and soil C cycling (Taylor, 1989; Hattenschwiler and Vitousek, 2000; Talbot and Treseder, 2012).

### DNA Extraction and Real-Time Quantitative PCR

The soil-litter samples after incubation were frozen for further DNA analysis. Following the manufacturer’s instructions, DNA was extracted using the MoBio PowerSoil DNA Isolation Kit (MOBIO Laboratories, United States). The V3–V4 region of bacterial 16S rRNA gene was selected and the primer 341F (5′-CCTAYGGGRBGCASCAG-3′) and 806R (5′-GGACTACNNGGGTATCTAAT-3′) was used to for PCR amplification. We used the primer ITS5-1737F (GGAAGTAAAAGTCGTAACAAGG) and ITS2-2043R (GCTGCGTTCTTCATCGATGC) for amplifications of ITS rRNA gene. The five replicates of PCR reactions were carried out in 30 μL mixture, containing 0.2 μM of forward and reverse primers, 10 ng template DNA, and 15 μL of Phusion^®^ High-Fidelity PCR Master Mix (New England Biolabs).

PCR amplification products were mixed in equal density ratios and conducted in 2% agarose gel using electrophoretic detection. We use GeneJET^TM^ Gel Extraction Kit (Thermo Scientific) to purify PCR mixture. Following the manufacturer’s instructions, a TruSeq DNA PCR-Free Sample Preparation Kit (Illumina, United States) was used to build sequencing libraries. Additionally, Qubit (Qubit 2.0 Fluorometer, Thermo Scientific) and Q- PCR (Agilent Bioanalyzer 2100) were used to assess the sequencing library.

The fiver replicates of genomic DNA were analyzed by the high-throughput barcoded Illumina HiSeq 2500 PE250 sequencing platform. According to the unique barcode, sequencing reads were assigned to different samples. The clean reads were obtained by aligned with the reference database (Silva database^1^) (Quast et al., 2012). Then we compared the UCHIME algorithm to the Gold Database,^2^ the method was used to align clean tags so as to determine chimera sequences. Specifically, the same OTUs were those that sequences were of ≥97% similarity. Based on the Greengene Database,^3^ we used the RDP (Ribosomal Database Project) (version 2.2) classifier algorithm (Wang et al., 2007) to classify the representative sequence for each OTU and finally annotate the microbial species. In addition, the MUSCLE software (version 3.8.31) was used to assign the phylogenetic relationships among microbial species.

### Statistical Analysis

Significant differences of treatments on soil mineralization, litter-released carbon, PE, priming efficiency, and alpha diversity and richness indices for microbial community were detected by one-way analysis of variance (ANOVA) followed by a lest significant difference (LSD) multiple comparison using SPSS version 17.0 (SPSS Inc., Chicago, IL, United States). The non-metric multidimensional scaling (NMDS) based on the Bray–Curtis dissimilarity matrices was performed to visualize the bacterial and fungal community structure.

The relative abundances of microbial species were significantly changed by the litter addition. Thus, we use linear discriminant analysis (LDA) effect size (LEfSe^4^) to classify the specific microbial taxa (Segata et al., 2011). Threshold of >4 and a *p*-value < 0.05 were shown. Further, the relationship between specific microbial abundance and the litter chemistry was tested by spearman correlations in the R package “vegan.”

Canonical Correspondence Analysis (CCA) was conducted to show associations between microbial composition and litter chemistry using R package “vegan.” The relationships among soil processes, litter chemistry, and specific microbial drivers were revealed by the Correlation Heatmap, which was performed by the R package “corrplot.”

Although Correlation Heatmap show the importance of different litter chemistry, there is a difficulty to confirm the accurate explanation of litter chemistry and microbial taxa to soil priming. The hierarchical multiple linear regression was thus aimed to extract the litter chemistry and specific microbial driver to PE. Models with lowest Akaike information criterion (AICc) were retained. Statistical analyses were performed using R software version 4.0.3 (R Development Core Team, 2014).

## Results

### Litter Chemical Traits

The results showed that the single-species litters differed chemically (Table 1). The N content varied among the four single-species litters, with the N concentration was higher in Cm (36.84 ± 0.14 mg g^–1^), followed by Ah (17.74 ± 0.15 mg g^–1^), and Ps (6.75 ± 0.17 mg g^–1^). Lignin concentration in Ah (160.37 ± 2.13 mg g^–1^) was higher than in Cm (132.94 ± 1.82 mg g^–1^) and Ps (146.68 ± 1.32 mg g^–1^), while the cellulose content in Ps (177.15 ± 0.92 mg g^–1^) was much higher than that in Cm litter (137.06 ± 1.77 mg g^–1^). Ss in Ah and Cm were 67 and 59% of those in Ps. Ct concentration in Cm was 47 and 10% of that in Ah and Ps, respectively. Contents of δ^13^C in single litters varied from −28.59 ± 0.022 to −27.93 ± 0.03‰, and they were distinctly different from δ^13^C content in soil (−16.54 ± 0.03‰).

**TABLE 1:** Initial litter chemical traits and δ^13^C of three single species and their mixtures.

Chemical traits	Ah	Cm	Ps	AhCm	AhPs	CmPs	AhCmPs
N	17.74 ± 0.15	36.84 ± 0.14	6.75 ± 0.17	28.48 ± 0.36	12.77 ± 0.18	23.75 ± 0.22	20.72 ± 0.07
C	441.59 ± 0.15	445.22 ± 0.71	410.33 ± 0.17	446.28 ± 0.53	426.07 ± 0.29	429.53 ± 0.69	431.04 ± 0.31
L	160.37 ± 2.13	132.94 ± 1.82	146.68 ± 1.32	152.35 ± 0.53	155.18 ± 0.94	145.51 ± 1.88	148.03 ± 0.78
Ce	138.33 ± 0.91	137.06 ± 1.77	177.15 ± 0.92	121.35 ± 0.68	167.32 ± 0.36	169.59 ± 1.27	172.08 ± 0.58
Ss	48.15 ± 0.42	42.27 ± 0.52	71.12 ± 0.37	41.31 ± 0.64	52.14 ± 0.17	50.34 ± 0.73	42.53 ± 0.76
phM	61.66 ± 0.61	36.76 ± 0.31	57.89 ± 1.20	37.78 ± 0.47	55.92 ± 1.12	30.76 ± 0.29	40.08 ± 0.96
phW	65.39 ± 0.24	25.39 ± 0.23	72.14 ± 0.57	38.88 ± 0.23	59.77 ± 0.74	42.37 ± 0.76	43.77 ± 0.58
Ct	18.93 ± 0.11	8.94 ± 0.24	88.98 ± 2.19	7.21 ± 0.33	34.97 ± 0.22	26.03 ± 0.05	15.40 ± 0.73
δ^13^C	−28.59 ± 0.02	−27.93 ± 0.03	−28.05 ± 0.03	−28.13 ± 0.09	−28.32 ± 0.04	−27.87 ± 0.07	−27.89 ± 0.07

*N, nitrogen; C, carbon; L, lignin; Ce, cellulose; Ss, soluble sugar; phM, methanol-soluble phenol; phW, water-soluble phenol; Ct, condensed tannins.*

### Soil Organic Carbon Mineralization, Litter Released C, Priming Effect, and Priming Efficiency

**FIGURE 1:**
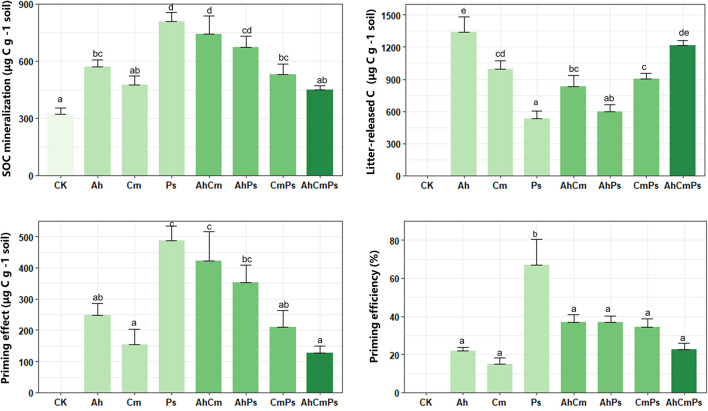
Soil organic matter mineralization, litter-released carbon, priming effect, and priming efficiency of after additions of single-species litters and litter mixtures. The different letters indicate the significant differences between litter addition treatments (*p* < 0.05). All data are presented as the mean ± SE (*n* = 5).

Compared with CK (321 ± 19.3 μg C g^–1^ soil), litter addition increased SOC mineralization, ranging from 447.8 ± 22.9 to 808.9 ± 44.87 μg C g^–1^ soil (Figure 1). Primed organic carbon contributed 48.8% to the soil-derived C, and priming efficiency was 14.86–66.90 μg C g^–1^ litter (Figure 1). Litter-released C ranged between 532.3 ± 74.3 and 1339.6 ± 140.6 μg C g^–1^ soil (Figure 1). Litter-released C across all samples was higher than SOC mineralization, and SOC mineralization in AhCmPs litter treatment was only 27% of total carbon mineralization (Supplementary Figure 1). The lowest SOC mineralization in AhCmPs litter treatment might because three litter species are mixed to form a more abundant nutrient gradient compared with single and two species, which can be better utilized by microorganisms, soil carbon degradation was thus low. Priming intensity and priming efficiency for Ps litter addition were much higher than for the Ah and Cm litter treatments (Figure 1).

### Microbial Abundance, Diversity, and Composition

**FIGURE 2:**
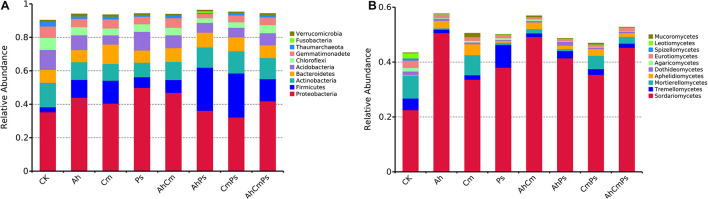
The relative abundance of phylotypes **(A)** in the bacterial community and **(B)** in the fungal community. Only taxa with average relative abundances >1% at each litter treatment are shown. All data are presented as the mean ± SE (*n* = 5).

We obtained a total of 9385 bacterial and 4236 fungal OTUs across all samples. The bacterial communities consisted mainly of *Proteobacteria* (42.3%), *Firmicutes* (11.3%), *Actinobacteria* (11.0%), *Acidobacteria* (8.8%), *Bacteroidetes* (8.5%), *Gemmatimonadetes* (5.0%), and *Chloroflexi* (4.8%) (Figure 2A and Supplementary Figure 2). The fungal community was dominated by *Sordariomycetes* (37.5%), *Mortierellomycetes* (3.0%), *Tremellomycetes* (2.1%), followed by *Aphelidiomycetes* (1.8%), *Eurotiomycetes* (1.0%), and *Mucoromycetes* (0.4%) (Figure 2B and Supplementary Figure 2).

**FIGURE 3:**
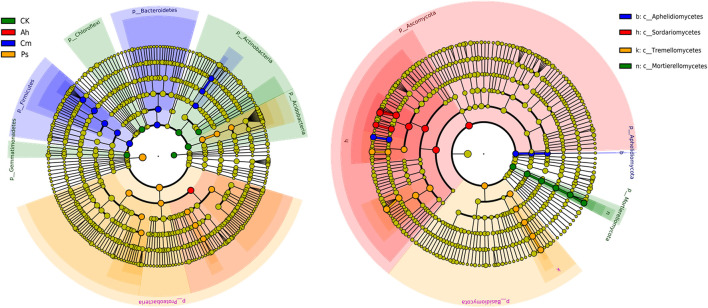
LEfSe analysis of bacterial community of the CK (no litter addition) and single litter addition treatments. A cladogram showing taxonomic representation of statistical and biological differences among groups. Taxa and nodes highlighted in different colors were significantly elevated in CK, Ah, Cm, and Ps litter addition, respectively (*p* < 0.01). Nodes remaining yellow indicate taxa that were not significantly different. The diameter of the circle is proportional to abundance.

Based on the LEfSe results, specific bacteria found predominantly in single litter additions belonged to phyla *Actinobacteria*, *Acidobacteria*, *Gemmatimonadetes*, and *Chloroflexi* in control, *Firmicutes* and *Bacteroidetes* in Cm and *Proteobacteria* in Ps litter addition treatment. The specific fungal classes were *Motierellomycetes*, *Eurotiomycetes* in CK, *Sordariomycetes* in Ah, *Aphelidiomycetes* in Cm, and *Tremellomycetes* in Ps (Figure 3).

**FIGURE 4:**
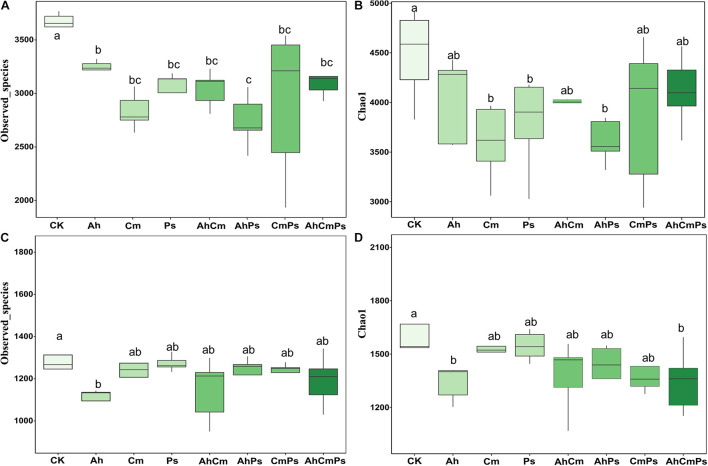
The effects of litter addition on soil bacterial and plant diversity. **(A)** Bacterial Observed_species, **(B)** bacterial Chao 1 diversity index, **(C)** fungal Observed_species, and **(D)** fungal Chao 1 diversity index. All data are presented as the mean ± SE (*n* = 5). Box plots show midline, median; box edges, first quartile and third quartile; and whiskers, minimum and maximum. The different letters indicate the significant differences between litter addition treatments (*p* < 0.05).

Litter addition markedly decreased bacterial Observed_speices (*F* = 4.223, *p* = 0.002), but not Chao 1 index (*F* = 2.069, *p* = 0.076). Bacterial Observed_speices was not different among the Cm, Ps, AhCm, CmPs, and AhCmPs treatments (Figures 4A,B). Observed_speices (*F* = 1.502, *p* = 0.202) and Chao 1 index (*F* = 1.694, *p* = 0.146) of fungal community were also not declined by litter addition. Fungal diversity was different between the control and Ah litter addition, but there was no difference among Cm, Ps, AhCm, AhPs, CmPs, and AhCmPs litter addition treatments (Figures 4C,D).

**FIGURE 5:**
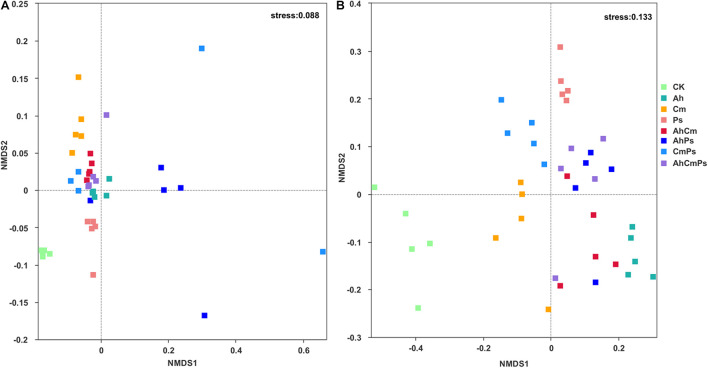
Non-metric multidimensional scaling (NMDS) analysis of **(A)** bacterial and **(B)** fungal community structures under different litter input.

Bacterial and fungal community composition were changed by litter addition. The stress score were 0.088 and 0.133, respectively (Figures 5A,B).

### Effects of Litter Chemistry and Microbial Taxa on Soil Processes

**FIGURE 6:**
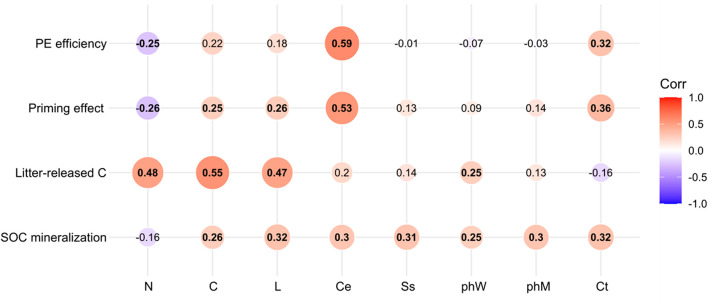
Correlation (*r*^2^) between litter chemical properties and SOC mineralization, litter-released C, priming effect, and priming efficiency. The numbers in the center indicate the correlation (Spearman’s *r* values) between litter chemistry and soil processes across all samples.

Soil organic carbon mineralization was influenced by all litter chemistry (all *p* < 0.05, *r*^2^ > −0.16) (Figure 6). There was a significant positive association between litter released-C and litter carbon (*p* < 0.01, *r*^2^ = 0.55), nitrogen (*p* < 0.01, *r*^2^ = 0.48), lignin (*p* < 0.01, *r*^2^ = 0.47), and phW (*p* < 0.05, *r*^2^ = 0.25). The PE was accelerated by cellulose concentration (*p* < 0.05, *r*^2^ = 0.53), litter Ct, carbon, and lignin, and negatively correlated with litter nitrogen (*p* < 0.05, *r*^2^ = −0.26). Litter with high concentration of cellulose efficiently primed native SOC mineralization (*p* < 0.05, *r*^2^ = −0.59) (Figure 6).

Although litter addition changed microbial alpha-diversity and community composition, soil priming intensity was not related to microbial diversity and community structure (Supplementary Figure 3).

**FIGURE 7:**
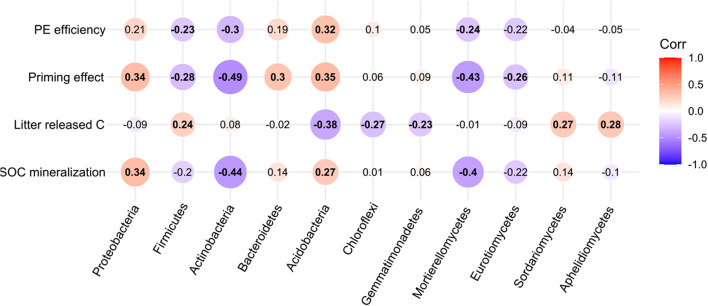
The effect of critical bacterial and fungal taxa on SOC mineralization, litter-released carbon, priming effect, and priming efficiency. The numbers in the center indicate the correlation (Spearman’s *r* values) between microbial taxa and soil processes across all samples.

Soil organic carbon mineralization increased with increasing *Proteobacteria* (*p* < 0.05, *r*^2^ = 0.34) and *Acidobacteria* (*p* < 0.05, *r*^2^ = 0.27) abundances, and decreased with *Actinobacteria* (*p* < 0.01, *r*^2^ = −0.44) abundance. Litter released carbon was negatively correlated with *Acidobacteria* (*p* < 0.01, *r*^2^ = −0.38), *Chloroflexi* (*p* < 0.05, *r*^2^ = −0.27), and *Gemmatimondetes* (*p* < 0.05, *r*^2^ = −0.23) abundance, and positively affected by *Firmicutes* (*p* < 0.05, *r*^2^ = 0.24). Priming intensity were significantly increased by *Proteobacteria* (*p* < 0.01, *r*^2^ = 0.34), *Acidobacteria* (*p* < 0.01, *r*^2^ = 0.35), *Bacteroidetes* (*p* < 0.01, *r*^2^ = 0.30), and reduced by *Firmicutes* (*p* < 0.05, *r*^2^ = −0.28) and *Actinobacteria* (*p* < 0.01, *r*^2^ = −0.49) abundances. At class level of fungal community, soil processes were mainly influenced by *Mortierellomycetes* (*p* < 0.05, *r*^2^ = −0.43), and by *Eurotiomycetes* (*p* < 0.05, *r*^2^ = −0.26). Litter released-C was positively increased by *Sordariomycetes* (*p* < 0.05, *r*^2^ = 0.27) and *Aphelidiomycetes* (*p* < 0.05, *r*^2^ = 0.28) (Figure 7).

### Responses of Microbial Taxa to Litter Chemistry

**FIGURE 8:**
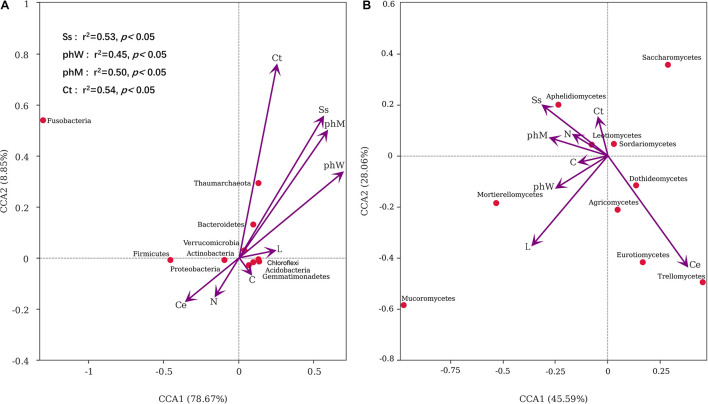
Canonical Correspondence Analysis plots showing the relationships between **(A)** dominant bacteria phyla, **(B)** dominant fungi classes and litter chemical properties. The significant influences of litter chemistry across all samples were labeled (*p* < 0.05).

Composition of the bacterial phylum was significantly influenced by litter Ss (*r*^2^ = 0.53, *p* = 0.01), phW (*r*^2^ = 0.45, *p* = 0.03), phM (*r*^2^ = 0.50, *p* = 0.01), and Ct (*r*^2^ = 0.54, *p* = 0.04) concentration. These variables could explain 87.52% of the total variance of bacterial composition with the first two canonical axes of the CCA model (Figure 8A). The first two axes also explained 73.65% of the variance in fungal composition, but fungal composition was not correlated to any of the litter chemistry variables (Figure 8B).

### Key Factors Influencing Soil Priming Intensity

The combination model of nitrogen and cellulose concentrations affected the PE directly (*r*^2^ = 0.279, *p* < 0.01, Table 2), while litter Ss, water-soluble phenol, methanol-soluble phenol and Ct indirectly influence soil priming intensity *via* variation to soil bacterial composition (Figure 8A). From the perspective of soil microbial community, PE was better to be explained by the abundance of critical bacterial and fungal taxa, such as *Proteobacteria*, *Firmicutes*, *Bacteroidetes*, *Acidobacteria*, and *Eurotiomycetes* (*r*^2^ = 0.41, *p* < 0.01) (Table 2).

**TABLE 2:** Outcome of determination of stepwise regressions between litter chemical traits and specific microbial taxa and soil priming intensity.

	Predictors (regression coefficients)	Strength (*r*^2^)	Significance (*p*)
Priming effect	N (−0.012**), Ce (0.001*)	0.279	2.82 × 10^–6^
	Proteobacteria (1.880*), Firmicutes (1.775*), Bacteroidetes (5.233***), Acidobacteria (7.270***), Eurotiomycetes (−4.7289*)	0.407	4.34 × 10^–7^

The strength (*r*^2^) and significance (*p*) of models with lowest AICc displayed. *P < 0.05; **P < 0.01; ***P < 0.001.

## Discussion

Our results confirmed our hypothesis (a) that priming induced by the addition of litter mixtures accelerated degradation of soil organic matter in an agro-forest-pastoral ecosystem. Further, we quantified that priming intensity varied significantly with litter chemistry from 24 to 74%. The results were partly inconsistent with hypothesis (b) in that the magnitude of priming was directly affected by litter macro-chemistry, but indirectly by litter micro-chemistry. Although SOC mineralization and litter decomposition was directly influenced by micro-chemistry (Ss, phW, phM, and Ct), the PE was not related to other tested microchemical variables, except Ct concentration. We have further shown that priming intensity was directly modified by critical microbial taxa, and litter nitrogen and cellulose concentration, but indirectly accelerated by litter micro-chemistry.

### Variation in Soil Priming Effect With Litter Chemistry

The litter and soil C processes were influenced by macronutrient and tannin contents. Litter decomposition significantly increased by litter nitrogen and carbon contents. It is interesting to note that lignin improved litter decomposition in this study, and that contradicted the traditional paradigm that lignin inhibits litter decomposition (Cotrufo et al., 2013). The positive role of lignin may result from light-absorbing compounds in lignin which may lead to preferential lignin degradation; it has been demonstrated that photochemical degradation of fresh residuals was important in semiarid areas (Austin and Ballare, 2010). In contrast to litter decomposition, soil C processes were accelerated by litter cellulose and tannin concentration. It has been shown that lignin has the potential to protect cellulose degradation (Talbot, 2012; Chao et al., 2019). Since photochemical mineralization of lignin leaves litter cellulose unshielded, it is degraded rapidly by extracellular enzymes at the expense of high nutrient requirement from SOM (Chen et al., 2020). Furthermore, we found that a large proportion of tannins corresponded to a high soil priming; this contrasted with a previous study suggesting that tannin before degradation can impede SOC decomposition because of an inhibitory effect on soil enzyme activity (Liu et al., 2017; Chao et al., 2019). The increase in the magnitude of priming with increasing tannin concentration could be due to the formation of protein complexes in the soil and that can explain why priming in our Ps treatment was relatively high (Kraus et al., 2004).

Composition of the dominant bacterial community was mainly shaped by litter micronutrients, which was because litter addition accompanied by sufficient labile compounds may have activated some bacterial taxa. Neither macronutrients nor micronutrients altered the composition of the dominant fungal community, which was probably due to the capacity of fungal taxa to utilize a wide spectrum of substrates (Wang et al., 2021).

Overall, these results showed that litter macronutrients affected soil processes and litter micronutrients altered bacterial community composition, but fungal community composition was not changed by litter chemistry. To determine which microbial taxa were influenced by the micro-nutrients and resulted in the soil-PE, we further explored responses of soil processes to specific bacterial and fungal taxa.

### Microbial Agents of Soil Priming Intensity

This study highlighted the specificity of various bacterial and fungal phylogenetic groups for the decomposition of different types of organic matter. Other studies have also shown that several microbial groups play a primary role in soil priming (Whitaker et al., 2014). For example, litter appeared to be first mineralized by *Firmicutes*. Our results are similar to other studies that used a DBA-SIP direct approach (Pascault et al., 2013). Indeed, *Firmicutes* was considered with strong catabolic capacities and usually responded rapidly to labile C (Wang et al., 2021). However, *Chloroflexi* and *Gemmatimondetes* were negatively correlated with litter released-C, indicating their low capacity to use labile C, which was primarily attributed to their oligotrophic nature (Ling et al., 2017). Similarly, it has been reported that *Chloroflexi* abundance was high in nutrient-poor soil (Fierer et al., 2012), while *Gemmatimondetes* was considered as SOM miners (Razanamalala et al., 2018); however, abundances of these groups in our study were not related to soil processes. The reason for the loss of these relationships might because the soil was sampled on a large scale since we were intended to provide a representative value that can be extrapolated to the farming-pastoral ecosystem (Montgomery et al., 2019).

Soil carbon was mainly utilized by *Proteobacteria* and *Bacteroidetes*. It has been suggested that all Proteobacteria subgroups are correlated with basal respiration (Razanamalala et al., 2018). Similar to our study, phylotypes of the *Bacteroidetes* have been previously implicated as cellulose consumers (Trivedi et al., 2013). Additionally, *Firmicutes* and *Acidobacteria* density was closely correlated with both litter decomposition and soil organic matter mineralization. It may be because they released extracellular enzymes to non-target degradation of SOM and partly decayed plant litter (Blagodatskaya et al., 2014). It has also been demonstrated that *Acidobacteria* were *k*-strategists which can explain priming intensity, and *Firmicutes* are known for their cellulolytic capacities (Pepe-Ranney et al., 2016; Sui et al., 2019). While some studies reported no change in *Actinobacteria* after nutrient addition (Li et al., 2016), abundance of *Actinobacteria* was increasing here after different litter addition, which might because their ability to compete with fungi for complex organic compounds and lignin-derived plant material (Boer et al., 2005; Di Lonardo et al., 2017; Liu X. et al., 2020).

The abundance of *Eurotiomycetes* were negatively correlated with soil priming, this was primarily attributed to their limited ability to degrade recalcitrant compounds and enhanced capacity for cellulose and sugar utilization (Osono, 2007). Additionally, *Mortierellomycetes* was seen to increase for high quality material, which was supposed to be response intensively to easily degradable material and was abundant in nitrogen-rich material (Clocchiatti et al., 2020; Liu et al., 2021).

## Conclusion

We demonstrated here that soil priming accounted for nearly half of SOC mineralization, indicating that priming should be incorporated into global land surface models to increase prediction accuracy of future SOC stocks in the context of global warming. The efficiency of priming induced by adding Ps litter was higher than that of other litters, showing the great influence of Ps planting on agricultural soil. Although litter addition changed microbial alpha-diversity and community composition, soil priming intensity was not related to microbial diversity and community structure. Litter decomposition and SOC mineralization were affected by both macro-chemistry and micro-chemistry, while priming intensity was only directly related to macro-chemistry and Ct concentration. Our study further illustrated that litter micro-chemistry indirectly affected soil priming *via* variation to bacterial community composition. Additionally, whereas priming intensity was directly controlled by litter nitrogen and cellulose concentration, critical microbial phylum *Proteobacteria*, *Firmicutes*, *Bacteroidetes*, *Acidobacteria*, and *Eurotiomycetes* class were the dominant drivers to soil priming.

## Data Availability Statement

The datasets presented in the study are deposited in the NCBI repository, accession number PRJNA758440.

## Author Contributions

YL conceived and designed the study based on discussions involving HY, JZ, ZN, and DH. HY, JZ, and DH performed the experiments. HY and SW analyzed the results. HY drafted the manuscript. All authors had a chance to review the manuscript before submission and contributed to discussion and interpretation of the data and contributed to the article and approved the submitted version.

## Conflict of Interest

The authors declare that the research was conducted in the absence of any commercial or financial relationships that could be construed as a potential conflict of interest.

## Publisher’s Note

All claims expressed in this article are solely those of the authors and do not necessarily represent those of their affiliated organizations, or those of the publisher, the editors and the reviewers. Any product that may be evaluated in this article, or claim that may be made by its manufacturer, is not guaranteed or endorsed by the publisher.

## Footnotes

1
https://www.arb-silva.de/

2
http://www.drive5.com/muscle/

3
https://greengenes.lbl.gov/Download/

4
http://huttenhower.sph.harvard.edu/lefse/
