# Supplementary material for: The Response of Critical Microbial Taxa to Litter Micro-Nutrients and Macro-Chemistry Determined the Agricultural Soil Priming Intensity After Afforestation
Source: Front Microbiol. 2021 Sep 16;12:730117. doi: 10.3389/fmicb.2021.730117 (PMC8481769; doi:10.3389/fmicb.2021.730117)
Supplement: Supplementary file 1 [file Data_Sheet_1.docx]

Fig S1. The proportion of litter-released C and SOC mineralization across all samples.


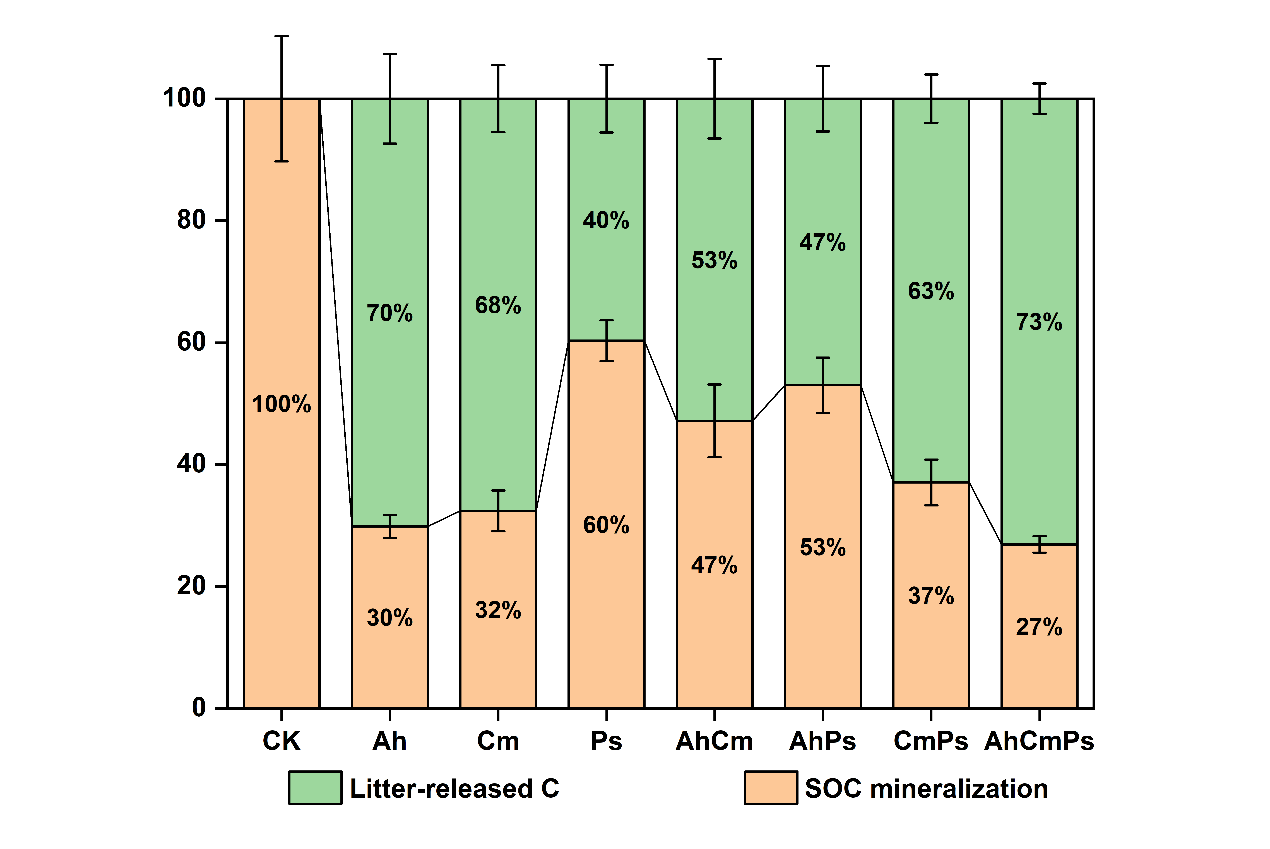


Fig S2. The relative abundance of bacterial phyla and fungal class across all litter addition treatments.

Fig S3. Correlations among microbial diversity and community structure and soil processes

SOC: SOC mineralization, Litter: litter released C, PE: priming effect,

Peeffi: PE efficiency, namds1_b&nmds2_b:bacterial community structure,

observed_b: observed_species in bacteria, Chao_b: bacterial chao diversity,

shannon_b: bacterial shannon diversity, simpson_b: bacterial simpson diversity,

observed_f: observed_species infungi, Chao_f: fungal chao diversity,

shannon_f: fungal shannon diversity, simpson_f: fungal simpson diversity,
